# Nsp1 facilitates SARS-CoV-2 replication through calcineurin-NFAT signaling

**DOI:** 10.1128/mbio.00392-24

**Published:** 2024-02-27

**Authors:** Wai-Yin Lui, Chon Phin Ong, Pak-Hin Hinson Cheung, Zi-Wei Ye, Chi-Ping Chan, Kelvin Kai-Wang To, Kit-San Yuen, Dong-Yan Jin

**Affiliations:** 1 School of Biomedical Sciences, The University of Hong Kong, Pokfulam, Hong Kong; 2 Department of Microbiology, The University of Hong Kong, Pokfulam, Hong Kong; 3 School of Nursing, Tung Wah College, Kowloon, Hong Kong; Virginia Polytechnic Institute and State University, Blacksburg, Virginia, USA

**Keywords:** SARS-CoV-2, Nsp1, calcineurin, NFAT, RCAN3, DDX5

## Abstract

SARS-CoV-2, the causative agent of COVID-19, has been intensely studied in search of effective antiviral treatments. The immunosuppressant cyclosporine A (CsA) has been suggested to be a pan-coronavirus inhibitor, yet its underlying mechanism remained largely unknown. Here, we found that non-structural protein 1 (Nsp1) of SARS-CoV-2 usurped CsA-suppressed nuclear factor of activated T cells (NFAT) signaling to drive the expression of cellular DEAD-box helicase 5 (DDX5), which facilitates viral replication. Nsp1 interacted with calcineurin A (CnA) to displace the regulatory protein regulator of calcineurin 3 (RCAN3) of CnA for NFAT activation. The influence of NFAT activation on SARS-CoV-2 replication was also validated by using the Nsp1-deficient mutant virus. Calcineurin inhibitors, such as CsA and VIVIT, inhibited SARS-CoV-2 replication and exhibited synergistic antiviral effects when used in combination with nirmatrelvir. Our study delineated the molecular mechanism of CsA-mediated inhibition of SARS-CoV-2 replication and the anti-SARS-CoV-2 action of calcineurin inhibitors.

**IMPORTANCE:**

Cyclosporine A (CsA), commonly used to inhibit immune responses, is also known to have anti-SARS-CoV-2 activity, but its mode of action remains elusive. Here, we provide a model to explain how CsA antagonizes SARS-CoV-2 through three critical proteins: DDX5, NFAT1, and Nsp1. DDX5 is a cellular facilitator of SARS-CoV-2 replication, and NFAT1 controls the production of DDX5. Nsp1 is a viral protein absent from the mature viral particle and capable of activating the function of NFAT1 and DDX5. CsA and similar agents suppress Nsp1, NFAT1, and DDX5 to exert their anti-SARS-CoV-2 activity either alone or in combination with Paxlovid.

## INTRODUCTION

The COVID-19 pandemic has taken millions of lives, disrupted the world economy, and segregated people and communities. As the causative agent of COVID-19, SARS-CoV-2 exhibits high transmissibility, immune evasion capability, and mutability to adapt to the host (1–3). Thanks to the joint efforts of the scientific community, multiple effective vaccines and therapeutics against SARS-CoV-2 have been developed. As a result of this and of the significantly reduced pathogenicity of the circulating strains of SARS-CoV-2, a rapid drop in mortality has been documented (4). Yet, SARS-CoV-2 continues to mutate and adapt to the vaccinated and infected human population, which makes the virus even more immunoevasive and transmissible. Despite the Omicron strains being much less virulent, the virus can hardly be eradicated from the human population (5).

Nsp1 is a crucial virulence factor of SARS-CoV-2, which is translated during the initial phase of viral replication (6). Nsp1 shuts off host mRNA translation and suppresses type I interferon response in favor of viral replication (7). It also augments viral mRNA translation by recognizing the 5′ untranslated region of the viral mRNA (8), which could also be bound by ribosomal protein S3 (RPS3) (9). Mechanistically, Nsp1 might enhance cap-independent translation of viral transcripts (10) and bi-directionally modulate RPS3 function (9). In addition, it could target cellular transcripts by inducing mRNA cleavage (11), binding to the 40S subunit to impede mRNA and eIF1A accommodation (12), preventing ribosome collision (13), inhibiting translation initiation (14), and promoting translation termination or degradation (9, 15). The knockout of Nsp1 was found to severely impair the replicability and viability of the virus, underlining the importance of this highly conserved viral protein (16). Several studies have generated SARS-CoV-2 mutants carrying K164A/H165A that impairs Nsp1’s translational shutoff capability, but they are viable and showed reduced or similar replicability compared to wild-type (WT) virus (17–19). A more detailed mutational analysis of Nsp1 revealed the importance of its N-terminal domain but the requirement of all domains for translational shutoff function (20, 21).

Meanwhile, β-coronaviruses such as SARS-CoV, MERS-CoV, and HCoV-OC43 have been shown to be inhibited by an immunosuppressant cyclosporine A (CsA) in a previous study (22). On the other hand, an elevated level of nuclear factor of activated T cells (NFAT) expression was seen in clinical samples of COVID-19 patients (23). These studies suggested that CsA, an inhibitor of NFAT, might suppress viral replication through the NFAT pathway. NFAT is a family of transcription factors, which critically regulate immunity, inflammation, angiogenesis, cellular proliferation, and differentiation (24). The NFAT proteins are controlled by upstream calmodulin-dependent serine/threonine phosphatase calcineurin. The change in cytosolic Ca^2+^ level leads to the formation of the calmodulin-Ca^2+^ complex, which interacts with calcineurin to induce NFAT dephosphorylations, thus activating NFAT-dependent transcription. This calmodulin-calcineurin-NFAT pathway is negatively regulated by the expression of regulator of calcineurin 3 (RCAN3), which prohibits the interaction between calcineurin proteins. The expression level of NFAT is another point for positive regulation (24). In addition, calcineurin is inhibited by the cyclophilin-cyclosporin complex (25). Cyclophilins are thought to play proviral roles by stabilizing the interaction between the receptor-binding domain of viral spike (S) protein and cellular ACE2 receptor (22, 26). However, the knockout of cyclophilins has recently been shown to have no effect on SARS-CoV-2 replication, suggesting that cyclophilins are unlikely the major cause of CsA-mediated suppression of coronaviruses (27). Till now, no in-depth and concrete investigation has been conducted to elucidate whether and how Nsp1 facilitates viral replication. Furthermore, the mechanism by which CsA mitigates viral replication remains elusive.

Here, we report that viral Nsp1 hijacks cellular NFAT signaling to facilitate SARS-CoV-2 replication. SARS-CoV-2 and Nsp1 were found to induce NFAT activation. The role of the NFAT pathway on SARS-CoV-2 replication was also studied using the S-deficient SARS-CoV-2 replicon and ΔNsp1 mutant virus. Dephosphorylation of endogenous NFAT1 was driven by the N terminal part of Nsp1. Nsp1 was also found to interact with calcineurin A (CnA), leading to the displacement of RCAN3 from CnA and the antagonization of RCAN3-mediated regulatory feedback on calcineurin activity. Elevated NFAT activity resulted in the upregulation of cellular DDX5, the knockdown of which abrogated Nsp1- or NFAT1-mediated SARS-CoV-2 replication enhancement. The anti-SARS-CoV-2 activity of calcineurin inhibitors such as CsA and VIVIT and their combination with SARS-CoV-2 main protease inhibitor nirmatrelvir was also assessed.

## RESULTS

### Activation of NFAT by SARS-CoV-2 and its Nsp1

CsA is a potent inhibitor for coronaviruses including SARS-CoV, MERS-CoV, and HCoV-HKU1 (22). Anti-SARS-CoV-2 activity of CsA has also been documented in cell models and clinical settings (28). However, the underlying mechanisms by which CsA mitigates SARS-CoV-2 infection remain largely unclear. CsA is an inhibitor of CnA, which is well studied for its regulatory role on NFAT to modulate innate immune response, T cell activation, and other cellular functions (27, 29). Considering together with the potential role of NFAT activation in SARS-CoV-2 replication (23), we hypothesized that the suppression of the CnA-NFAT signaling by CsA would show anti-SARS-CoV-2 activity.

**Fig 1:**
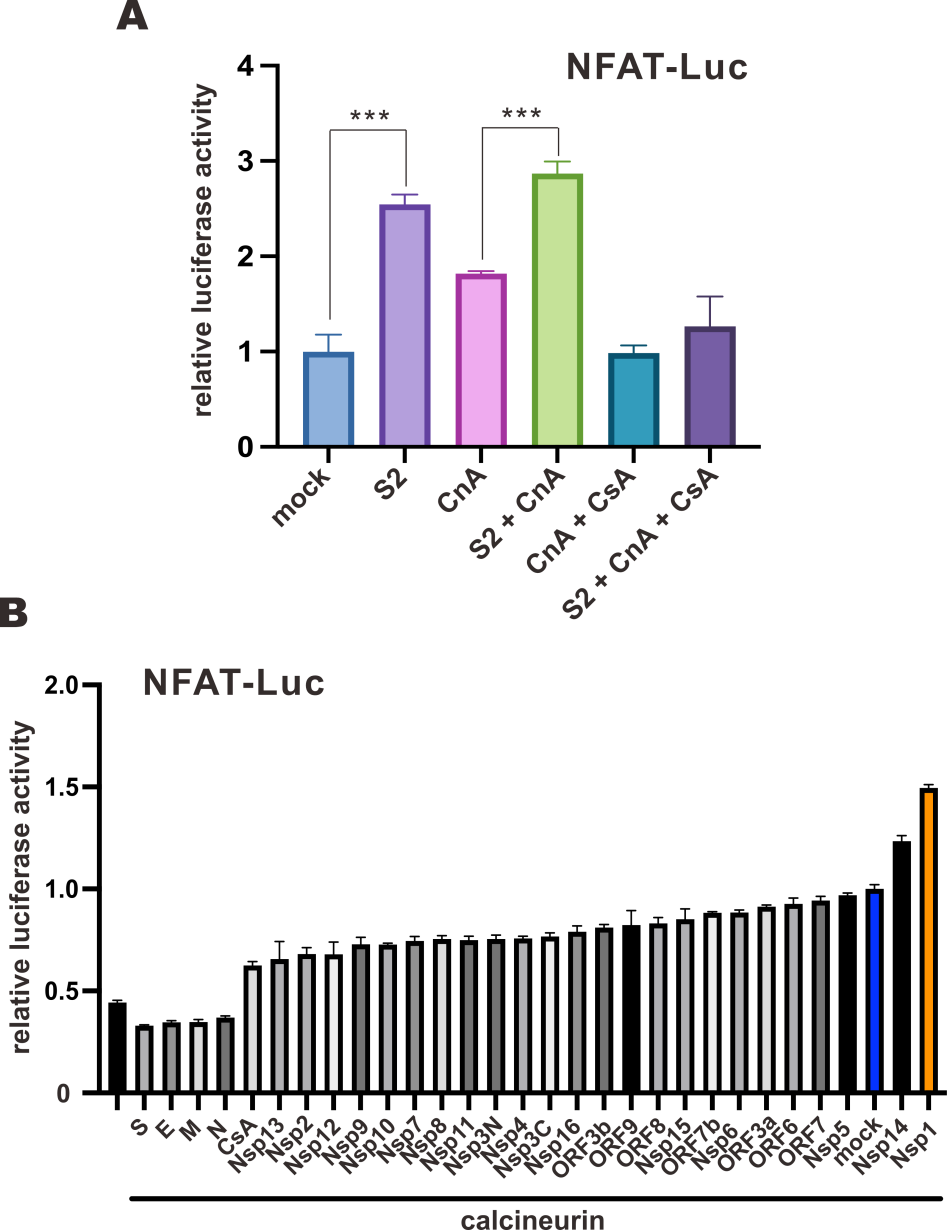
Induction of NFAT activation by SARS-CoV-2. (**A**) VeroE6-TMPRSS2 cells were either mock transfected with an empty vector or transfected with a firefly luciferase reporter plasmid driven by 3× NFAT promoter (NFAT-Luc), a control *Renilla* luciferase reporter plasmid driven by thymidine kinase promoter (TK-Luc), and FLAG-CnA expression plasmid 24 hours prior to infection with WT SARS-CoV-2 (S2) at an M.O.I. of 0.01. Cells were treated with 1 µM CsA after infection. Cells were harvested for dual luciferase assay 16 hours later. (**B**) HEK293T cells were either mock transfected with an empty vector or transfected with NFAT-Luc, TK-Luc, FLAG-CnA expression plasmid, and SARS-CoV-2 protein expression library for 24 hours. CsA (1 µM) was added to cells for 1 hour to serve as the negative control. Cells were harvested for dual luciferase assay. The mean values of three biological replicates (*n* = 3) were represented by the bars and their respective standard deviations were depicted as error bars. The statistical significance for the difference between the indicated groups was analyzed using a two-tailed Student’s *t*-test for paired samples, and the ranges of the *P* values were indicated (*P < 0.05; **P < 0.01; and ***P < 0.001).

When NFAT-Luc-transfected VeroE6-TMPRSS2 cells were infected with SARS-CoV-2, an increase in luciferase activity was observed in SARS-CoV-2-infected groups both in the absence and presence of CnA, but not in the presence of CsA (Fig. 1A). This result indicated that the infection of SARS-CoV-2 sufficiently drove NFAT activation. We next screened for the potential virulence factor(s) capable of inducing the activation of NFAT. A functional screen was performed in HEK293T cells using NFAT-Luc and an expression library of all SARS-CoV-2 proteins to study the effect of each viral protein on the NFAT pathway. NFAT-Luc was activated by CnA, and individual SARS-CoV-2 protein was overexpressed. Among SARS-CoV-2 viral proteins, enforced expression of Nsp1 and Nsp14 further elevated CnA-induced NFAT-Luc activity, while structural proteins including S, E, M, and N demonstrated inhibitory effect on NFAT-Luc (Fig. 1B). Since the largest increase in NFAT-Luc activity was spotted in Nsp1-expressing cells and the highly conserved coronaviral Nsp1 homologs have been shown to be a major virulence factor contributing to CsA-mediated inhibition of SARS-CoV and MERS-CoV (22, 30), Nsp1 of SARS-CoV-2 was selected for further investigations.

### Verification of the effect of Nsp1 with SARS-CoV-2 infection and replicon

**Fig 2:**
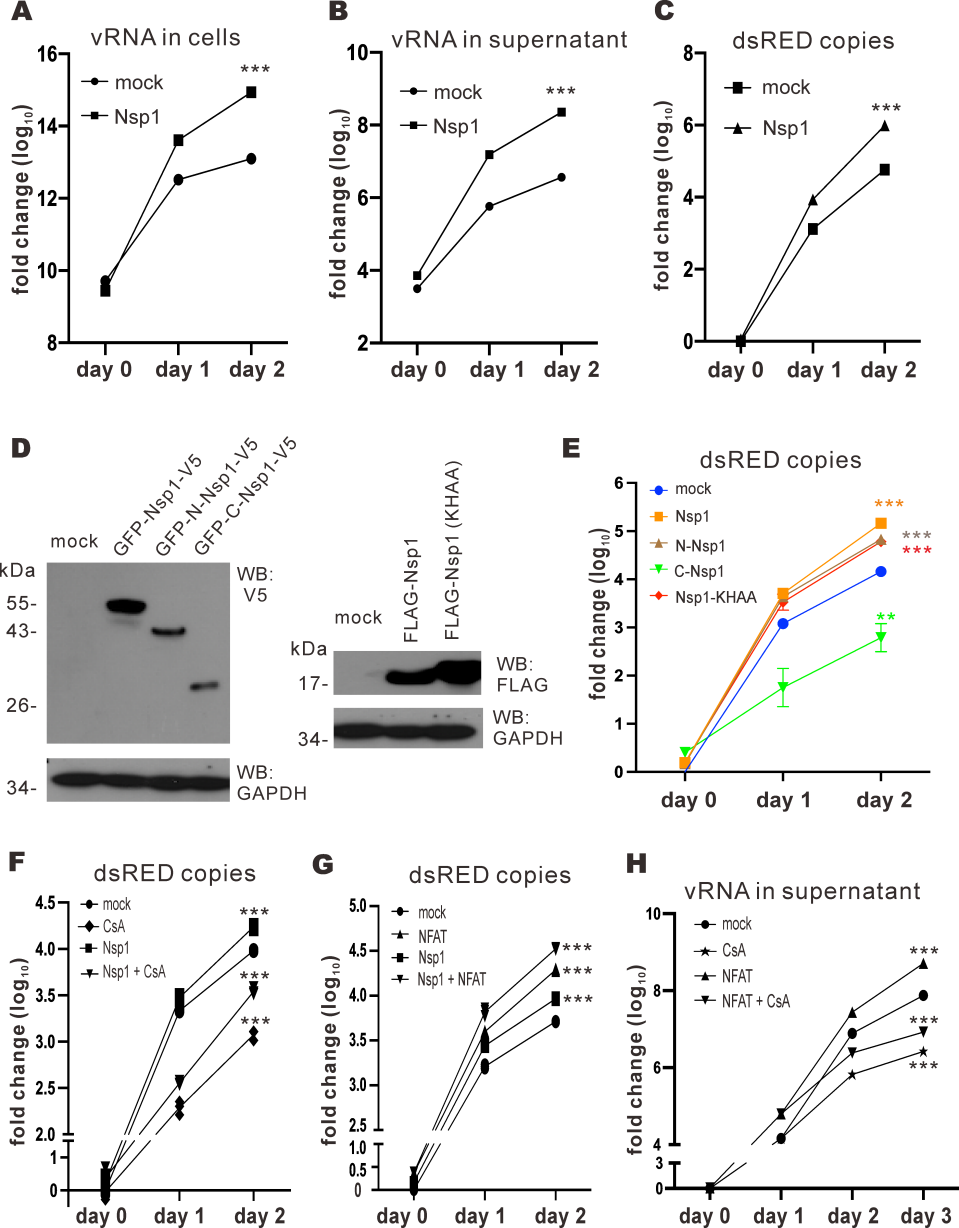
Nsp1 facilitates SARS-CoV-2 replication. (**A and B**) VeroE6-TMPRSS2 cells were either mock transfected with an empty plasmid or transfected with Nsp1 expression plasmid 24 hours before infection with WT SARS-CoV-2 at an M.O.I. of 0.1. Cell lysates and supernatants were harvested at the specified time points, and viral RNA in these samples was quantified by RT-qPCR. (**C**) VeroE6-TMRPSS2 cells were transfected with S-deficient SARS-CoV-2 replicon bacmid (ΔS replicon) plus either empty plasmid (mock) or Nsp1 expression plasmid. Cell lysates were harvested at the specified time points, and vRNA was quantified by RT-qPCR. (**D**) GFP-Nsp1-V5, GFP-N-Nsp1-V5, GFP-C-Nsp1-V5, FLAG-Nsp1, FLAG-Nsp1-KHAA, and empty plasmid (mock) were expressed in VeroE6-TMPRSS2 cells. Cell lysates were harvested after 24 hours of transfection. Nsp1 proteins were detected by anti-V5 or anti-FLAG. Endogenous glyceraldehyde 3-phosphate dehydrogenase (GAPDH) was used for normalization. (**E**) VeroE6-TMPRSS2 cells were transfected with ΔS replicon, GFP-Nsp1-V5, GFP-N-Nsp1-V5, GFP-C-Nsp1-V5, FLAG-Nsp1, and FLAG-Nsp1-KHAA expression plasmids and empty plasmid (mock). Cell lysates were harvested at the specified time points for the quantification of vRNA by RT-qPCR. (**F**) VeroE6-TMPRSS2 cells were transfected with ΔS replicon, Nsp1 expression plasmid, and empty plasmid (mock). Cells were treated with 1 µM CsA 6 hours post-transfection. Cell lysates were harvested at the specified time points for RT-qPCR. (**G**) VeroE6-TMPRSS2 cells were transfected with ΔS replicon, Nsp1 and NFAT1 expression plasmids, and empty plasmid (mock). Cell lysates were harvested at the specified time points for RT-qPCR. (**H**) VeroE6-TMPRSS2 cells were transfected with ΔS replicon and NFAT1 expression plasmids and empty plasmid (mock). After 24 hours, 1 µM CsA was added to the cells. Cell lysates were harvested at the specified time points for RT-qPCR. The mean values of three biological replicates (*n* = 3) were represented by the bars and their respective standard deviations were depicted as the error bars. The statistical significance for the difference between the indicated group and the mock was analyzed using a two-tailed Student’s *t*-test for paired samples, and the ranges of the *P* values were indicated (*P < 0.05; **P < 0.01; and ***P < 0.001).

The impact of Nsp1 on SARS-CoV-2 replication was studied. Nsp1 was overexpressed in VeroE6-TMPRSS2 cells prior to SARS-CoV-2 infection. Since Nsp1 is known to antagonize interferon production (16, 18, 31), the interferon-deficient VeroE6-TMPRSS2 cells were employed to study the effect of Nsp1 on SARS-CoV-2 replication. Viral RNA (vRNA) in the lysate and supernatant was harvested on days 0, 1, and 2 and measured by RT-qPCR (Fig. 2A and B). When Nsp1 was overexpressed, more vRNA copies were found in both cell lysate and supernatant on days 1 and 2 (Fig. 2A and B). To further scrutinize the effect of Nsp1 on SARS-CoV-2 replication specifically, an S-deficient SARS-CoV-2 replicon (ΔS replicon) was employed (32). The ΔS replicon was transfected into VeroE6-TMPRSS2 cells, and vRNA samples were harvested on days 0, 1, and 2 for RT-qPCR of dsRED gene copies. The SARS-CoV-2 S gene was replaced by dsRED so the amount of dsRED RNA can represent genomic copies of the ΔS replicon. For the mock group, increasing dsRED RNA was found from day 0 to day 2, consistent with the replication of the ΔS replicon in VeroE6-TMPRSS2 cells. Higher dsRED gene copies detected upon the expression of Nsp1 (Fig. 2C) indicated that Nsp1 facilitates SARS-CoV-2 replication.

To delineate the protein domain(s) of Nsp1 responsible for the facilitation of SARS-CoV-2 replication (17, 20, 21), Nsp1 mutants were constructed and expressed in VeroE6-TMPRSS2 cells together with the ΔS replicon. From the replication kinetics of ΔS, overexpression of Nsp1, N-Nsp1, and Nsp1-KHAA resulted in elevated replicability, while C-Nsp1 overexpression suppressed replication (Fig. 2E). Notably, since Nsp1-KHAA has been reported to have lost its ability to inhibit host translation (14), the enhanced replicability of the ΔS replicon for the Nsp1-KHAA-expressing group indicated that the observed Nsp1-mediated replication enhancement might not be ascribed to host translational shutoff. Altogether, these results were compatible with the N terminal part of Nsp1 responsible for the enhanced SARS-CoV-2 replication.

To investigate how Nsp1 might affect CsA-mediated suppression of viral replication, Nsp1 was overexpressed in the ΔS replicon-transfected cells before they were treated with CsA. The CsA-treated group had fewer dsRED gene copies from day 0 to day 2, suggesting an inhibition of ΔS replication (Fig. 2F). Opposite to this, overexpression of Nsp1 alongside CsA treatment showed higher dsRED gene copies (Fig. 2F), indicating a partial rescue of ΔS replication from CsA-mediated suppression by Nsp1.

Considering that CsA treatment would result in the suppression of the NFAT pathway, the role of NFAT in SARS-CoV-2 replication was investigated in the context of Nsp1 expression. When NFAT1 or Nsp1 was expressed with the ΔS replicon, more dsRED gene copies were detected (Fig. 2G). Coexpression of Nsp1 and NFAT1 led to a further increase in dsRED gene copies (Fig. 2G), indicating an additive effect of NFAT1 and Nsp1 in facilitating viral replication. We next studied the impact of CsA treatment on NFAT1-dependent enhancement of SARS-CoV-2 replication. A higher vRNA level was detected on day 1 when NFAT1 was overexpressed (Fig. 2H). After CsA was added on day 1, a marked decrease in vRNA amount was observed in the CsA-only group on days 2 and 3, whereas the NFAT1 + CsA group showed a higher vRNA level than the CsA-only group (Fig. 2H). Thus, NFAT1-mediated replication enhancement was only partially rescued by CsA treatment. Since CsA inhibits calcineurin activity but does not directly affect NFAT activation, the partial reduction of ΔS replicability might be attributed to compromised calcineurin and activated NFAT1. Hence, NFAT activation would facilitate SARS-CoV-2 replication, while CsA-mediated suppression of ΔS replication can be partially reversed by Nsp1 overexpression.

### Facilitation of ΔNsp1 replication by supplementation of NFAT1 *in trans*

**Fig 3:**
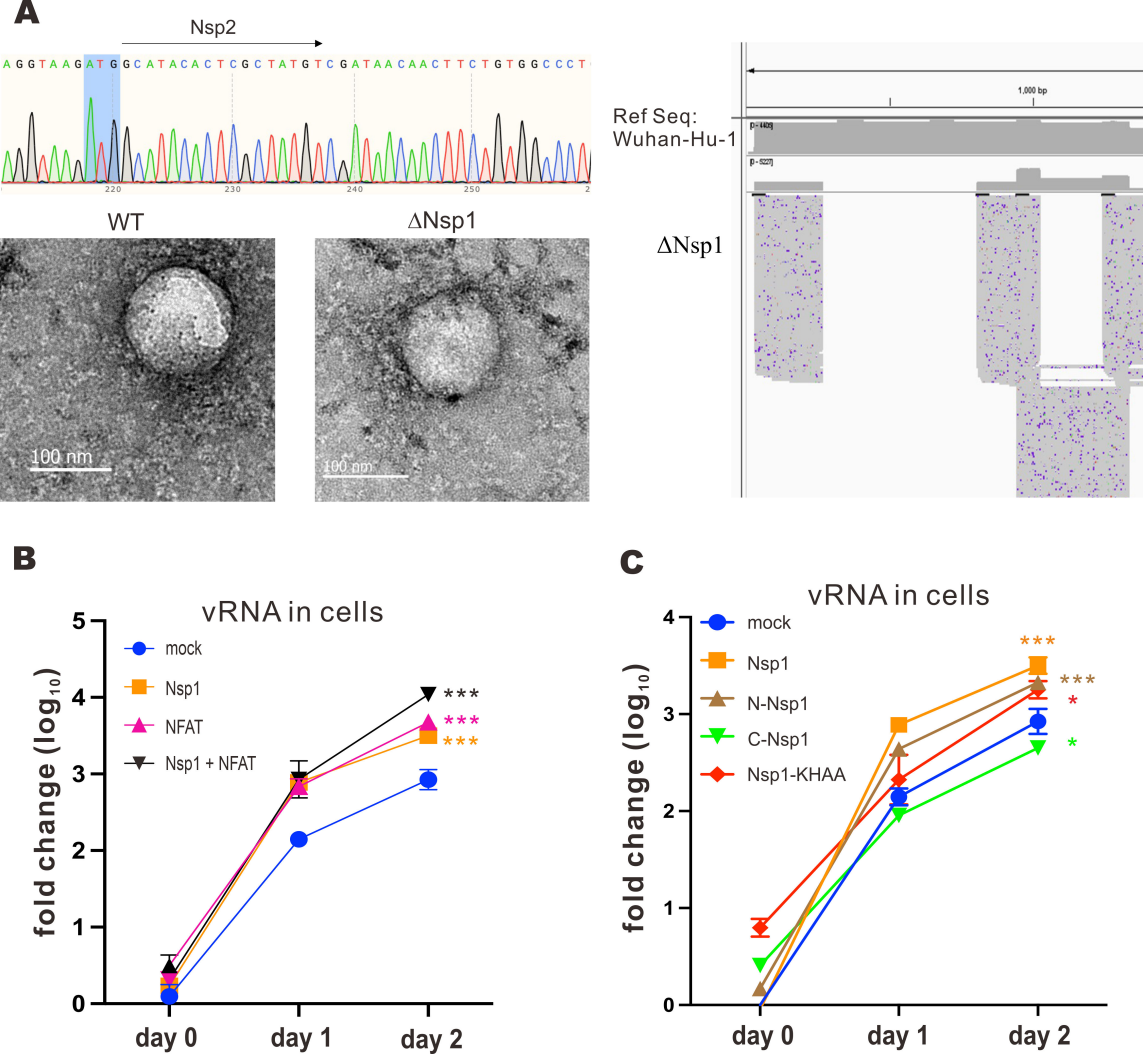
Construction of ΔNsp1 bacmid and replication kinetics of ΔNsp1 virus. (**A**) ΔNsp1 bacmid was constructed using BAC recombineering. The DNA sequence was confirmed by Sanger sequencing. The ΔNsp1 bacmid was transfected into HEK293T cells using Genejuice. After 4 days, the supernatant was harvested. The produced ΔNsp1 was fixed with 4% paraformaldehyde and scanned using transmission electron microscopy. Viral mRNA was also harvested for nanopore sequencing. SARS-CoV-2 Wuhan Hu-1 parental strain served as the reference sequence. Viral transcripts expressed from Wuhan Hu-1 and ΔNsp1 strains were then compared to reveal differentially expressed genes. (**B**) VeroE6-TMPRSS2 cells were transfected with Nsp1 and NFAT1 expression plasmids, as well as an empty plasmid (mock), 24 hours prior to infection with ΔNsp1 at an M.O.I. of 0.01. Cell lysates were harvested at the specified time points, and the vRNA was quantified with RT-qPCR. (**C**) VeroE6-TMPRSS2 cells were transfected with GFP-Nsp1-V5, GFP-N-Nsp1-V5, GFP-C-Nsp1-V5, FLAG-Nsp1, and FLAG-Nsp1-KHAA expression plasmids and empty plasmid (mock) 24 hours prior to ΔNsp1 infection at an M.O.I. of 0.01. Cell lysates were harvested at the specified time points, and vRNA was quantified with RT-qPCR. The mean values of three biological replicates (*n* = 3) were represented by the bars, and their respective standard deviations were depicted as the error bars. The statistical significance for the difference between the indicated group and the mock was analyzed using a two-tailed Student’s *t*-test for paired samples and the ranges of the *P* values were indicated (*P < 0.05; **P < 0.01; and ***P < 0.001).

In order to determine how NFAT activation might affect SARS-CoV-2 replication in connection to its interplay with Nsp1 protein, an Nsp1-deficient SARS-CoV-2 infectious clone was constructed via BAC recombineering. The bacmid was sequenced to ensure correct deletion of the complete Nsp1-coding region (Fig. 3A), before being transfected into HEK293T cells to produce Nsp1-deficient SARS-CoV-2, denoted as ΔNsp1 hereafter. The presence and morphology of ΔNsp1 virion in the supernatant were confirmed with transmission electron microscopy (TEM), with WT SARS-CoV-2 virus serving as the positive control (Fig. 3A). To further ensure the genomic integrity of the mutant virus, the whole viral mRNA transcriptome was sequenced by nanopore sequencing. From the nanopore sequencing result, only Nsp1 mRNA was missing in the ΔNsp1 transcriptome, while all other viral mRNAs were expressed properly (Fig. 3A). ΔNsp1 infection demonstrated a much weaker cytopathic effect than WT SARS-CoV-2 infection in VeroE6-TMPRSS2 cells. These results verified the successful generation of a ΔNsp1 bacmid and recombinant virus.

ΔNsp1 was then employed to study the influence of NFAT activation on its replication in relation to the interplay between Nsp1 and NFAT. VeroE6-TMPRSS2 cells expressing Nsp1, NFAT1, and Nsp1 + NFAT1 were infected with ΔNsp1. vRNA in the cell lysates was harvested on days 0, 1, and 2 for RT-qPCR analysis. For the mock group, more vRNA was observed from day 0 to day 2, indicating replication of ΔNsp1 (Fig. 3B). When cells were expressing Nsp1, NFAT1, or Nsp1 + NFAT1, more vRNA copies were detected than the mock group, suggesting that overexpression of Nsp1 and/or NFAT facilitates ΔNsp1 replication. Notably, the coexpression of both proteins resulted in a more substantial enhancement of viral replication (Fig. 3B). On the other hand, Nsp1 mutants were expressed in VeroE6-TMPRSS2 cells infected with ΔNsp1. Among the Nsp1 mutants, Nsp1, N-Nsp1, and Nsp1-KHAA were associated with more vRNA copies, consistent with more robust ΔNsp1 replication, while overexpression of C-Nsp1 led to diminished vRNA level (Fig. 3C). Hence, the N terminal part of Nsp1 might play an important role in facilitating ΔNsp1 replication.

### RCAN3-mediated suppression of viral replication rescued by Nsp1 overexpression

**Fig 4:**
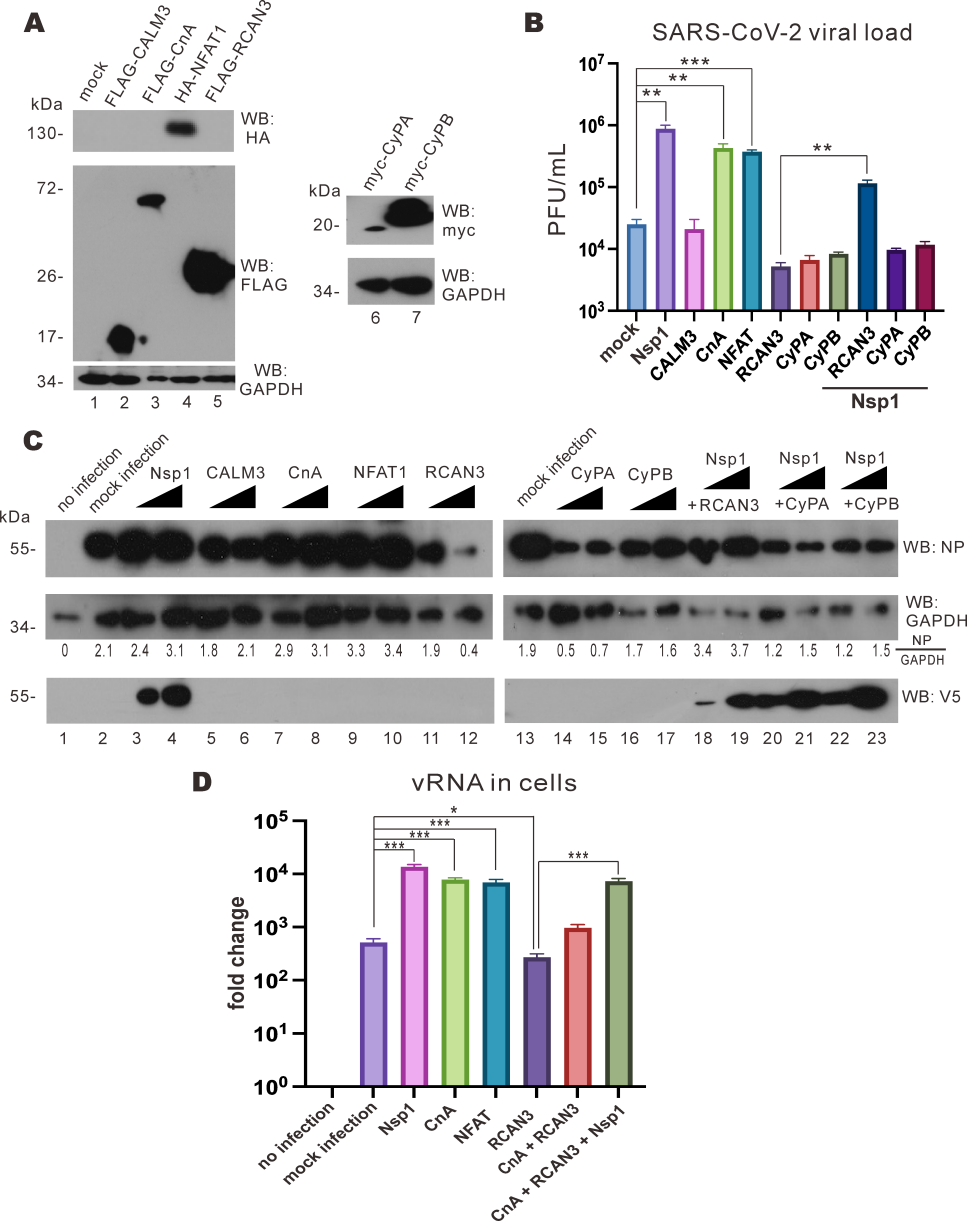
Nsp1 overexpression rescues RCAN3-mediated suppression of SARS-CoV-2 replication. (**A**) VeroE6-TMPRSS2 cells were transfected with FLAG-CALM3, FLAG-CnA, HA-NFAT1, FLAG-RCAN3, Myc-CyPA-His, and Myc-CyPB-His expression plasmids. Cell lysates were harvested after 24 hours for Western blotting with anti-HA, anti-FLAG, anti-Myc, and anti-GAPDH antibodies. (**B**) VeroE6-TMPRSS2 cells were transfected with Nsp1, FLAG-CALM3, FLAG-CnA, HA-NFAT1, FLAG-RCAN3, Myc-CyPA-His, and Myc-CyPB-His expression plasmids 24 hours prior to infection with SARS-CoV-2 at an M.O.I. of 0.1 for plaque assay. (**C**) VeroE6-TMPRSS2 cells were transfected with V5-Nsp1, FLAG-CALM3, FLAG-CnA, HA-NFAT1, FLAG-RCAN3, Myc-CyPA-His, and Myc-CyPB-His expression plasmids 24 hours prior to wild-type SARS-CoV-2 infection at an M.O.I. of 0.1. After 1 hour of incubation, the viral culture medium was replaced with Dulbecco’s modified Eagle medium (DMEM) solution with 2% FBS. After 24 hours post-infection, the cell lysates were harvested for Western blotting with anti-SARS-CoV-2 N protein (NP), anti-V5, and anti-GAPDH antibodies. SARS-CoV-2 NP and glyceraldehyde 3-phosphate dehydrogenase (GAPDH) band densities were quantified by ImageJ, and the NP/GAPDH ratios were calculated. (**D**) VeroE6-TMPRSS2 cells were transfected with V5-Nsp1, FLAG-CALM3, FLAG-CnA, HA-NFAT1, FLAG-RCAN3, Myc-CyPA-His, and Myc-CyPB-His expression plasmids and empty plasmid 24 hours prior to ΔNsp1 infection at an M.O.I. of 0.01. After 1 hour of incubation, the viral culture medium was replaced with DMEM solution with 2% FBS. After 24 hours, cell lysates were harvested for viral RNA analysis by RT-qPCR. The mean values of three biological replicates (*n* = 3) were represented by the bars, and their respective standard deviations were depicted as the error bars. The statistical significance for the difference between the indicated groups was analyzed using a two-tailed Student’s *t*-test for paired samples, and the ranges of the *P* values were indicated (*P < 0.05; **P < 0.01; and ***P < 0.001).

To study how effector proteins in the NFAT signaling pathway might affect SARS-CoV-2 replication, calmodulin 3 (CALM3), CnA, NFAT1, regulator of calcineurin family protein 3 , cyclophilin A (CyPA), and cyclophilin B (CyPB) were expressed in VeroE6-TMPRSS2 cells (Fig. 4A). Cyclophilins have previously been suggested to be the proviral host factors for SARS-CoV-2 replication since antiviral effect was seen when cells were treated with drugs that inhibit cyclophilins (22). In our experiment, higher viral titers measured by plaque assay were observed upon expression of Nsp1, CnA, and NFAT1, whereas the opposite trend was evident in cells expressing RCAN3, CyPA, and CyPB (Fig. 4B). This suggested that the activation of NFAT1 through the CnA axis might be responsible for the enhancement of SARS-CoV-2 replication. To confirm this, an increasing dose of these effector proteins was expressed in VeroE6-TMPRSS2 cells infected with SARS-CoV-2, and the cell lysates were harvested to determine the levels of SARS-CoV-2 nucleocapsid (N) protein. Increasing the expression of Nsp1, CnA, and NFAT led to a more pronounced detection of SARS-CoV-2 N protein (Fig. 4C, lanes 3, 4, and 7–10). On the contrary, N protein expression was diminished when increasingly more RCAN3, CyPA, and CyPB were produced (Fig. 4C, lanes 11, 12, and 14–17). Interestingly, when Nsp1 was coexpressed with RCAN3, SARS-CoV-2 N protein expression was more prominent (Fig. 4C, lanes 18 and 19), while no observable change in SARS-CoV-2 N protein expression was noticed while increasing the expression of Nsp1 with CyPA or CyPB (Fig. 4C, lanes 20–23). These results suggested that NFAT activation would facilitate SARS-CoV-2 replication, whereas NFAT regulatory proteins such as RCAN3, CyPA, and CyPB would block SARS-CoV-2 replication. Notably, Nsp1 can relieve RCAN3-mediated suppression of SARS-CoV-2 replication but not CyPA- or CyPB-mediated suppression.

To further delineate the mechanism by which NFAT1 activation facilitates ΔNsp1 replication, effector proteins in the NFAT pathway were expressed in VeroE6-TMPRSS2 cells infected with ΔNsp1. Cell lysates were harvested at 2 days post-infection for RT-qPCR analysis of vRNA. Whereas the expression of Nsp1, CnA, and NFAT1 led to an increase in vRNA levels, less vRNA was detected in cells expressing RCAN3, but the amount of vRNA was restored when Nsp1 was coexpressed with RCAN3 (Fig. 4D). This result further supported the role of NFAT1 activation in ΔNsp1 replication and an interplay between RCAN3 and Nsp1.

### Induction of NFAT dephosphorylation by Nsp1

**Fig 5:**
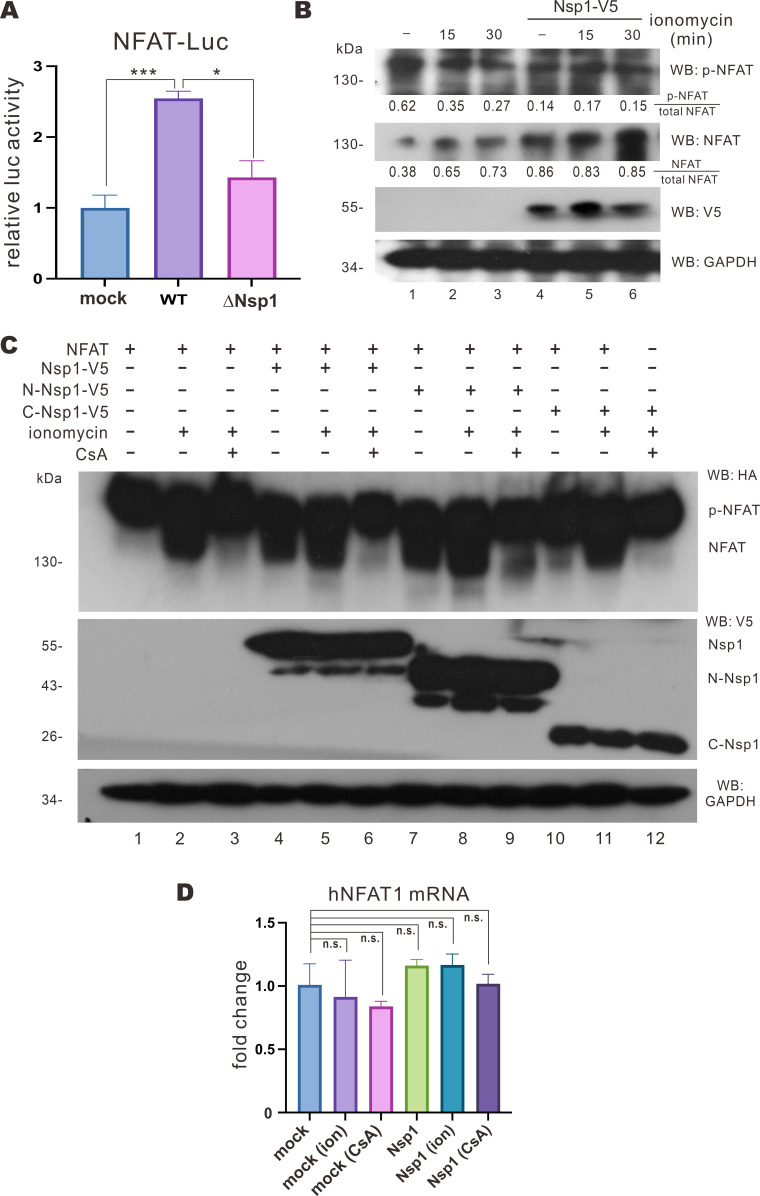
Nsp1 induces NFAT1 dephosphorylation. (**A**) VeroE6-TMRPSS2 cells were transfected with NFAT-Luc and TK-Luc 24 hours prior to WT SARS-CoV-2 and ΔNSP1 infection at an M.O.I. of 0.01. Cell lysates were harvested for dual luciferase assay. luc, luciferase. (**B**) VeroE6-TMPRSS2 cells were transfected with GFP-Nsp1-V5 expression plasmid and treated with 2 µM ionomycin 24 hours post-transfection for the indicated period. Cell lysates were harvested and analyzed by Western blotting with anti-phospho-NFAT, anti-NFAT, anti-V5, and anti-GAPDH antibodies. p-NFAT and NFAT band densities were quantified by ImageJ, and the p-NFAT/total NFAT (p-NFAT + NFAT) and NFAT/total NFAT ratios were derived. (**C**) HEK293T cells were transfected with GFP-Nsp1-V5, GFP-N-Nsp1-V5, GFP-C-Nsp1-V5, and HA-NFAT1 expression plasmids for 24 hours and then treated with 1 µM CsA, 2 µM ionomycin, and 10 µM calcium chloride solution for 30 min. Cell lysates were harvested and analyzed by Western blotting with anti-HA, anti-V5, and anti-GAPDH antibodies. (**D**) HEK293T cells were transfected with empty plasmid (mock) or GFP-Nsp1-V5 expression plasmid and then treated with 1 µM CsA and 2 µM ionomycin (ion) for 30 min before collecting cellular mRNA for RT-qPCR. The mean values of three biological replicates (*n* = 3) were represented by the bars and their respective standard deviations were depicted as the error bars. The statistical significance for the difference between the indicated groups was analyzed using a two-tailed Student’s *t*-test for paired samples, and the ranges of the *P* values were indicated (*P < 0.05; **P < 0.01; ***P < 0.001; and n.s., not significant).

To shed more mechanistic light on the impact of Nsp1 on NFAT activation, cells were transfected with NFAT-Luc and TK-Luc 24 hours prior to infection with WT SARS-CoV-2 and mutant ΔNsp1 viruses at an M.O.I. of 0.01. A significant increase in NFAT-Luc activity was seen in cells infected with WT SARS-CoV-2, whereas only weak induction of NFAT-Luc activity was observed in ΔNsp1-infected cells (Fig. 5A). Thus, Nsp1 is a major driver of NFAT activation in the early phase of SARS-CoV-2 infection. The cellular NFAT1 and phospho-NFAT levels were then determined. Treatment with ionomycin, a calcium ionophore that activates NFAT (24), for 15 and 30 min led to an increase in NFAT1 level and a decrease in phospho-NFAT1 level (Fig. 5B, lanes 2 and 3 vs 1). When Nsp1 was expressed, a marked rise in NFAT level and a fall in the amount of phospho-NFAT were noticed (Fig. 5B, lane 4 vs 1). Thus, Nsp1 might induce NFAT1 dephosphorylation. When Nsp1 was expressed, treatment with ionomycin for 15 or 30 min failed to further induce NFAT1 dephosphorylation (Fig. 5B, lanes 5 and 6 vs 4). This suggested that Nsp1 expression would drive NFAT dephosphorylation potently.

To further investigate the protein domain(s) of Nsp1 required for the observed dephosphorylation event, Nsp1, N-Nsp1, and C-Nsp1 mutants were expressed in HEK293T cells treated with calcium + ionomycin in the presence and absence of CsA. When cells that did not express any Nsp1 mutant were treated with calcium + ionomycin, a slow-migrating HA-NFAT1 moiety was seen in the immunoblot, consistent with dephosphorylation (Fig. 5C, lane 2 vs 1). When the same cells were treated with calcium chloride + ionomycin + CsA, the observed band shift of HA-NFAT1 disappeared, suggesting the inhibition of dephosphorylation (Fig. 5C, lane 3 vs 2). The band shift of HA-NFAT1 was seen upon expression of Nsp1 or N-Nsp1 (Fig. 5C, lane 4 or 7 vs 1) but disappeared in the presence of C-Nsp1 (Fig. 5C, lane 10 vs 1). Meanwhile, no statistically significant change in the total mRNA levels of NFAT1 was noticed (Fig. 5D). These results suggested that the N terminal part of Nsp1 is sufficient for the induction of NFAT1 dephosphorylation, which is susceptible to inhibition by CsA.

### Binding of Nsp1 to CnA and consequent displacement of RCAN3

**Fig 6:**
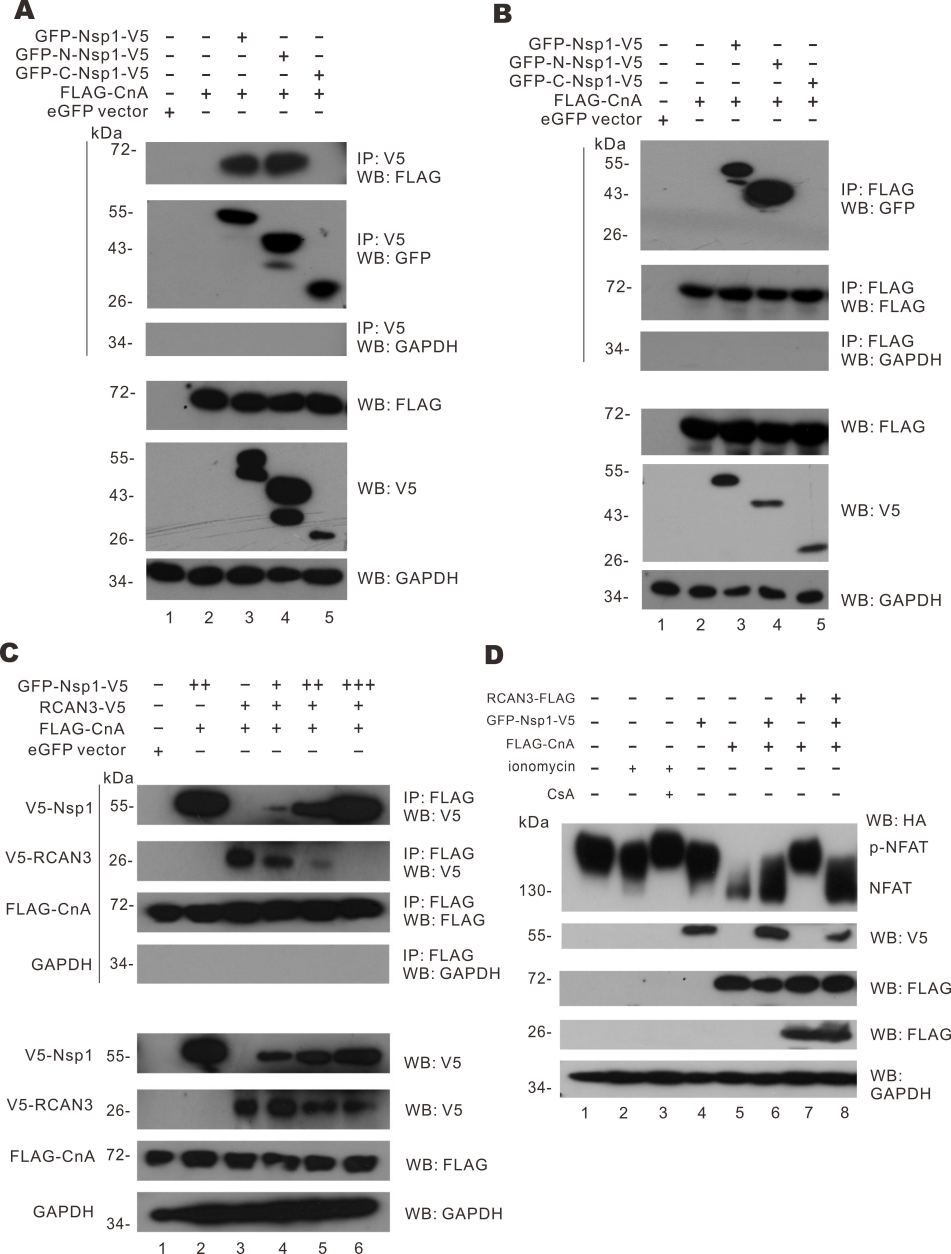
Mitigation of RCAN3-mediated NFAT activation by Nsp1. (**A**) HEK293T cells were transfected with eGFP-C1 empty vector, FLAG-CnA, GFP-Nsp1-V5, GFP-N-Nsp1-V5, and GFP-C- Nsp1-V5 expression plasmids for 24 hours. Cell lysates were harvested for immunoprecipitation using anti-V5 antibodies. The immunoprecipitates and cell lysates were analyzed by Western blotting with anti-GFP, anti-FLAG, anti-V5, and anti-GAPDH antibodies. (**B**) HEK293T cells were transfected with eGFP-C1 empty vector, FLAG-CnA, GFP-Nsp1-V5, GFP-N-Nsp1-V5, and GFP-C-Nsp1-V5 expression plasmids for 24 hours. Cell lysates were harvested for immunoprecipitation using anti-FLAG antibodies. The immunoprecipitates and cell lysates were analyzed by Western blotting with anti-GFP, anti-FLAG, anti-V5, and anti-GAPDH antibodies. (**C**) HEK293T cells were transfected with eGFP-C1 empty vector, FLAG-CnA, GFP-Nsp1-V5, and V5-RCAN3 expression plasmids for 24 hours. Cell lysates were harvested for immunoprecipitation with anti-FLAG. The immunoprecipitates and cell lysates were analyzed by Western blotting with anti-FLAG, anti-V5, and anti-GAPDH antibodies. (**D**) HEK293T cells were transfected with eGFP-C1 empty vector, HA-NFAT, FLAG-CnA, GFP-Nsp1-V5, and FLAG-RCAN3 expression plasmids for 24 hours. Cells were treated with 1 µM CsA, 2 µM ionomycin, and 10 µM calcium chloride solution for 30 min before harvest and analyzed by Western blotting with anti-HA, anti-V5, anti-FLAG, and anti-GAPDH antibodies.

Since Nsp1 is not known to have catalytic activity, we hypothesized that the effect of Nsp1 on NFAT activation would primarily be mediated through direct interaction with effector protein(s). Co-immunoprecipitation experiments were carried out between Nsp1 and the effector proteins including CALM3, CnA, and NFAT1. Whereas none to very slight interaction was found between Nsp1 and CALM3 or NFAT1, strong bands of FLAG-tagged CnA were detected in the immunoprecipitates of Nsp1 and N-Nsp1 (Fig. 6A, lanes 3 and 4), while no band of FLAG-CnA can be observed in the immunoprecipitates of C-Nsp1 (lane 5). Reciprocal co-immunoprecipitation was performed to confirm the interaction between CnA and Nsp1 or N-Nsp1. From the immunoprecipitates of FLAG-CnA, Nsp1 and N-Nsp1 were found but C-Nsp1 was absent (Fig. 6B). This demonstrated that Nsp1 binds to CnA via its N-terminal domain.

Furthermore, the reversal of RCAN3-mediated suppression of SARS-CoV-2 replication by Nsp1 and a regulatory role of RCAN3 on CnA activity led us to the hypothesis that Nsp1 interacts with CnA to undermine the binding of RCAN3 to CnA, thus relieving its suppression on CnA activity. To substantiate this model, another co-immunoprecipitation experiment was set up. FLAG-CnA, V5-RCAN3, and increasing levels of V5-Nsp1 were expressed in HEK293T cells. FLAG-CnA was pulled down with anti-FLAG and the amount of V5-RCAN3 and V5-Nsp1 in the precipitate was examined. When CnA and RCAN3 were present, V5-RCAN3 in the FLAG-CnA complex was more pronounced (Fig. 6C, lane 3). With increasing doses of V5-Nsp1 in the cells, decreasing amounts of RCAN3 were detected in the FLAG-CnA precipitates (Fig. 6C, lanes 4–6). Thus, Nsp1 competes with RCAN3 to bind to CnA. With this competitive binding, Nsp1 hinders the regulatory action of RCAN3 on CnA.

We next assessed how the competition between Nsp1 and RCAN3 for binding with CnA might influence NFAT1 activation. CnA was expressed in HEK293T cells to induce NFAT1 activation (Fig. 6D, lane 5). As expected, the expression of Nsp1 triggered NFAT1 dephosphorylation (Fig. 6D, lanes 4 and 6). When RCAN3 was also expressed, a potent suppression of CnA-mediated NFAT1 dephosphorylation was observed (Fig. 6D, lane 7), but this was rescued by Nsp1 overexpression (Fig. 6D, lane 8). This result demonstrated that Nsp1 can rescue NFAT1 dephosphorylation from RCAN3-mediated suppression.

### Induction of DDX5 expression by Nsp1 and NFAT

**Fig 7:**
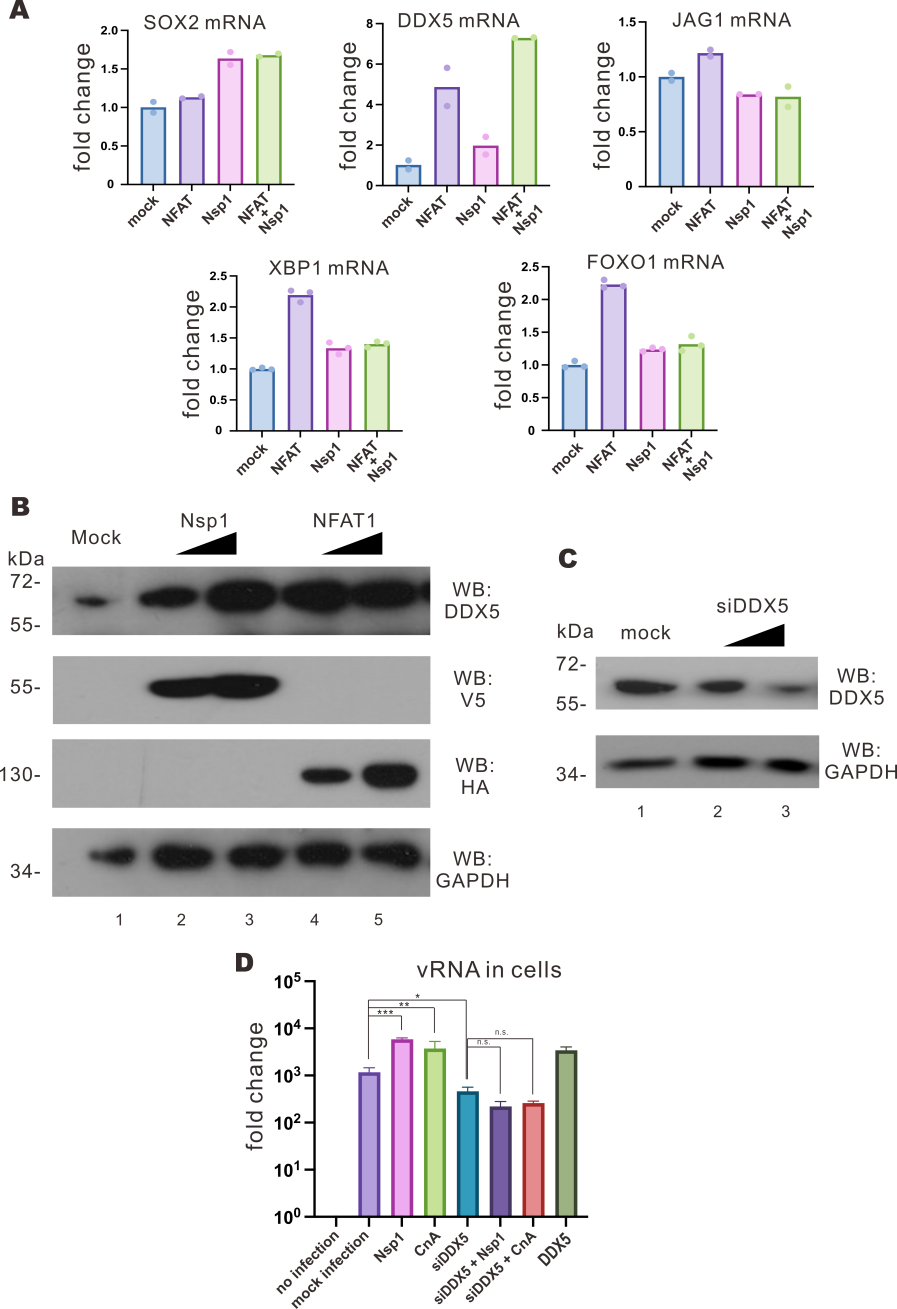
Induction of DDX5 expression by Nsp1 and NFAT1. (**A**) VeroE6-TMPRSS2 cells were transfected with Nsp1 and NFAT1 expression plasmids. Cell lysates were harvested 24 hours post-transfection for RT-qPCR. (**B**) VeroE6-TMPRSS2 cells were transfected with increasing doses of V5-Nsp1 and HA-NFAT1 expression plasmids. Cell lysates were harvested 24 hours post-transfection for Western blotting with anti-DDX5, anti-V5, and anti-HA antibodies. Endogenous glyceraldehyde 3-phosphate dehydrogenase (GAPDH) was used for normalization. (**C**) VeroE6-TMPRSS2 cells were transfected with increasing doses of siDDX5 or negative control. Cell lysates were harvested 24 hours post-transfection for Western blotting with anti-DDX5. Endogenous GAPDH was used for normalization. (**D**) VeroE6-TMPRSS2 cells were transfected with empty plasmid, Nsp1, CnA, and DDX5 expression plasmids and siDDX5 or negative control 24 hours prior to ΔNsp1 infection at an M.O.I. of 0.01. Cell lysates were harvested after 2 days post-infection. The statistical significance for the difference between the indicated groups was analyzed using a two-tailed Student’s *t*-test for paired samples, and the ranges of the *P* values were indicated (*P < 0.05; **P < 0.01; and ***P < 0.001).

We continued to investigate how the activation of NFAT might contribute to SARS-CoV-2 replication. With reference to the transcriptomic data in Nsp1-expressing lung cells in public databases, several upregulated genes that are known to be activated by NFAT1, including SOX2, DDX5, XBP1, FOXO1, JAG1, and BCL3, were selected and their expression levels in Nsp1- or NFAT1-expressing cells were validated as described (33). Nsp1 and NFAT1 were overexpressed in VeroE6-TMPRSS2 cells, and the expression levels of these transcripts were determined by RT-qPCR. Among them, the expression of DDX5 was elevated upon overexpression of NFAT1, Nsp1, and NFAT1 + Nsp1 (Fig. 7A). DDX5 is a DEAD-box helicase 5 with RNA-binding activity and is responsible for cell proliferation and organ development (34). DDX5 has previously been shown to be a proviral host factor that facilitates SARS-CoV-2 replication (35). The knockdown of DDX5 markedly suppressed SARS-CoV-2 replication putatively through an interaction with viral N protein. In addition to a direct interaction with SARS-CoV-2 N protein, DDX5 was also known to be a negative regulator of innate immunity. DDX5 was found to block p65- and DHX58-mediated antiviral responses triggered by RNA virus infection (36). With this in mind, we examined DDX5 expression in VeroE6-TMRPSS2 cells overexpressing Nsp1 or NFAT1. The increase of DDX5 in cells expressing Nsp1 or NFAT1 (Fig. 7B) suggested that Nsp1 and NFAT1 can induce the expression of DDX5 in VeroE6-TMRPSS2 cells.

To shed light on biological significance, DDX5 was knocked down by siRNA in ΔNsp1-infected VeroE6-TMPRSS2 cells that expressed increasing doses of Nsp1 and CnA. The knockdown of DDX5 was confirmed by Western blotting (Fig. 7C), and the replication of ΔNsp1 was examined. For ΔNsp1 infection, when siDDX5 was transfected, a slight decrease in vRNA level was found (Fig. 7D). When Nsp1 or CnA were expressed in the DDX5-knockdown cells, no increase in vRNA level was observed. In contrast, the overexpression of Nsp1 and CnA resulted in an increase in vRNA levels when DDX5 was not knocked down (Fig. 7D). Thus, the knockdown of DDX5 would counteract the effect of Nsp1 or CnA overexpression on SARS-CoV-2 replication, indicating that Nsp1 or CnA facilitates SARS-CoV-2 replication by upregulating DDX5 expression.

### Anti-SARS-CoV-2 activity of VIVIT

**Fig 8:**
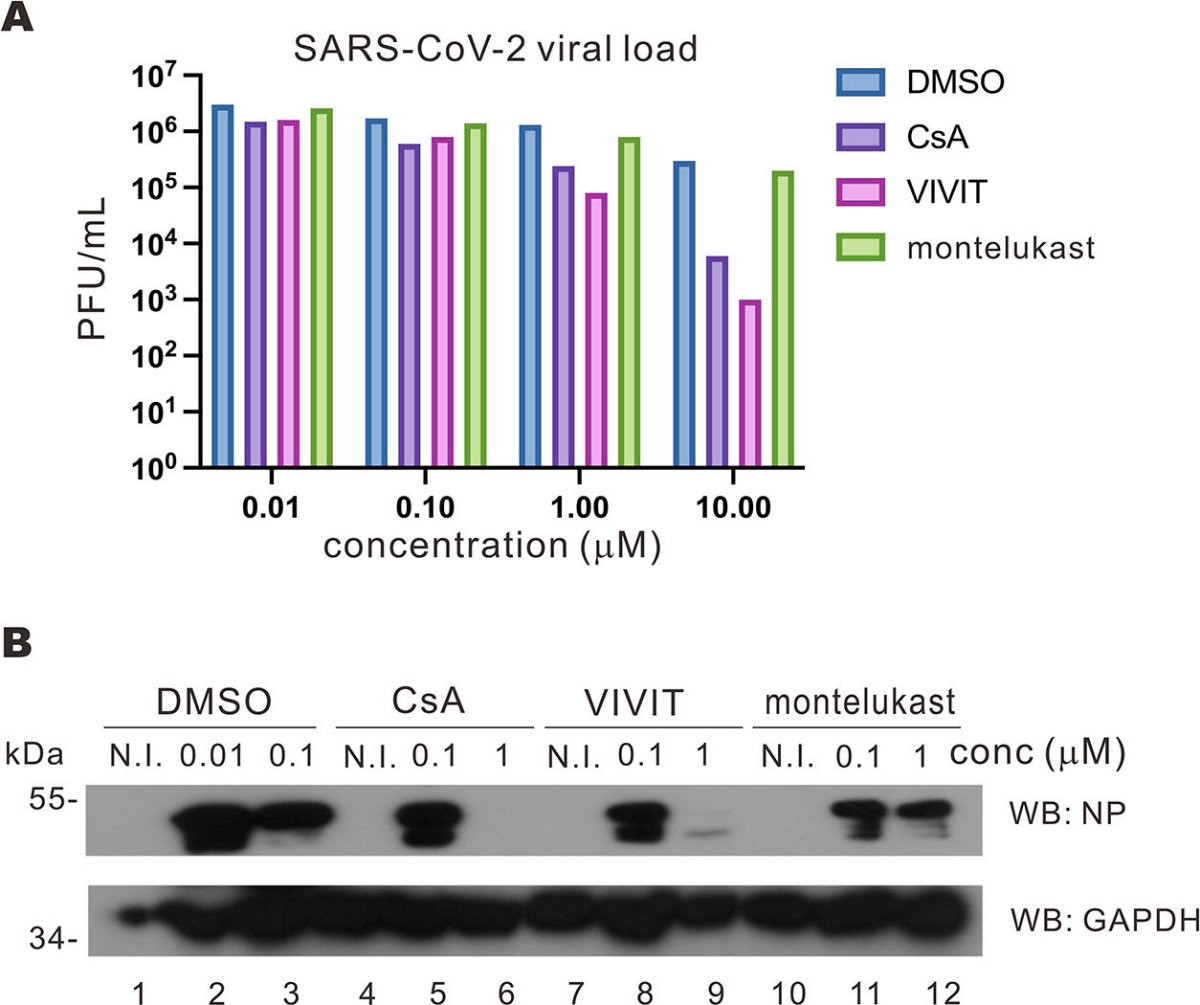
CnA inhibitors demonstrated antiviral activity against SARS-CoV-2. (**A**) Calu-3 cells were inoculated with SARS-CoV-2 at an M.O.I. of 0.1 for 1 hour, and the viral medium was replaced with Dulbecco’s modified Eagle medium (DMEM) with 2% FBS and the indicated drugs. After 3 days, supernatants were harvested to infect VeroE6-TMPRSS2 cells for plaque assay. (**B**) Calu-3 cells were inoculated with SARS-CoV-2 at an M.O.I. of 0.1 for 1 hour, and the viral medium was replaced with DMEM with 2% FBS and the indicated drugs. N.I. indicates no infection. At 3 days post-infection, cell lysates were harvested for Western blotting with anti-SARS-CoV-2 N protein (NP) and anti-GAPDH antibodies. conc, concentration.

The link of NFAT signaling to SARS-CoV-2 replication provides new antiviral strategies and lead compounds. To this end, we set out to test whether CsA and CnA-NFAT1 interaction blocker 11R-VIVIT (37), hereafter abbreviated as VIVIT, might inhibit SARS-CoV-2 replication. VIVIT was selected since we observed that Nsp1 facilitates SARS-CoV-2 replication by altering CnA activity, so the prohibition of CnA-NFAT interaction may mitigate such Nsp1-mediated induction without abolishing the functions of CnA, which are important for immune responses and other cellular processes (24, 38). We also include montelukast since it was predicted by a computational model to be an effective antagonist of Nsp1 (39). Calu-3 cells were infected with SARS-CoV-2 and then treated with the captioned drugs. The viral titer in the supernatant of the infected Calu-3 cells was then quantified with plaque assay. When Calu-3 cells were treated with CsA and VIVIT at or above 1 µM, a significant reduction in viral titer was observed, suggesting that both CsA and VIVIT had antiviral activity at concentrations of 1 µM or higher (Fig. 8A). No observable effect was observed for cells treated with CsA or VIVIT at less than 0.1 µM (Fig. 8A). The Calu-3 cell lysates were then harvested to check for viral protein expression. When Calu-3 cells were infected with SARS-CoV-2, N protein was abundantly detected (Fig. 8B). Treatment of cells with 1 µM CsA or VIVIT, but not montelukast, led to a remarkable reduction in N protein (Fig. 8B). These results demonstrated the anti-SARS-CoV-2 activity of calcineurin inhibitors.

### Synergistic antiviral effect of a combination of nirmatrelvir and calcineurin inhibitor

**Fig 9:**
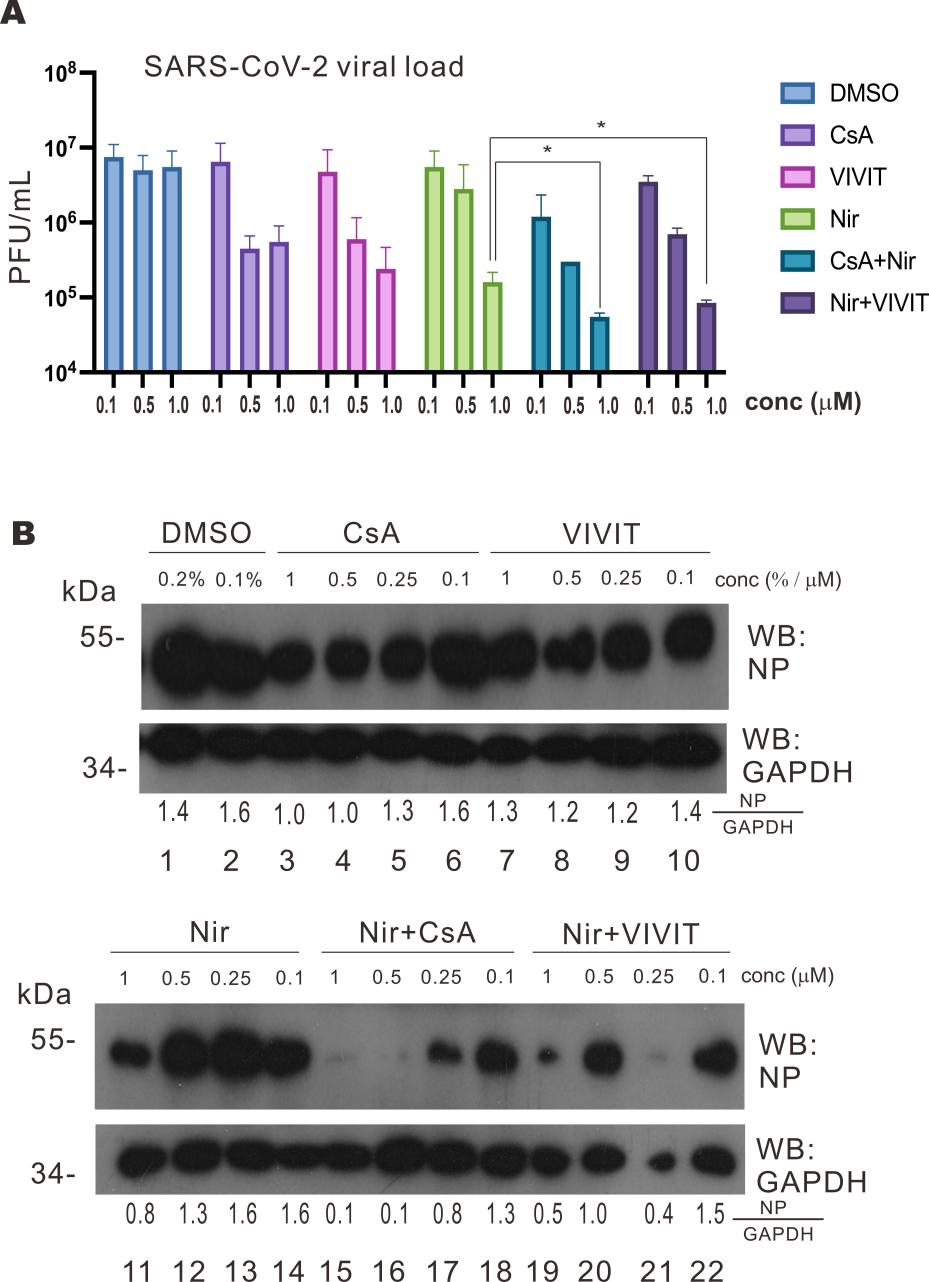
Combination of nirmatrelvir and calcineurin inhibitors shows synergistic antiviral effect. (**A**) Calu-3 cells were inoculated with SARS-CoV-2 at an M.O.I. of 0.1 for 1 hour, and the viral medium was replaced with Dulbecco’s modified Eagle medium (DMEM) with 2% FBS and the indicated drugs. For CsA + Nir and Nir + VIVIT, an equal concentration of drugs was added. After 3 days, supernatants were harvested to infect VeroE6-TMPRSS2 cells for plaque assay. conc, concentration. (**B**) Calu-3 cells were inoculated with SARS-CoV-2 at an M.O.I. of 0.1 for 1 hour, and the viral medium was replaced with DMEM with 2% FBS and the indicated drugs. After 3 days, cell lysates were harvested for Western blotting with anti-SARS-CoV-2 NP and anti-GAPDH antibodies. NP and glyceraldehyde 3-phosphate dehydrogenase (GAPDH) band densities were quantified by ImageJ, and the NP/GAPDH ratios were calculated. The mean values of three biological replicates (*n* = 3) were represented by the bars and their respective standard deviations were depicted as the error bars. The statistical significance for the difference between the indicated groups was analyzed using a two-tailed Student’s *t*-test for paired samples, and the ranges of the *P* values were indicated (*P < 0.05; **P < 0.01; and ***P < 0.001). Nir, nirmatrelvir.

Although calcineurin inhibitors demonstrated antiviral activity against SARS-CoV-2, it was not sufficiently strong to mitigate SARS-CoV-2 replication at low dose (1 µM). In this regard, it will be of interest to see whether a combination of calcineurin inhibitor with SARS-CoV-2 main protease inhibitor nirmatrelvir might produce a synergistic effect. Calcineurin inhibitors are commonly used as immunosuppressants for organ transplant patients to suppress graft rejection responses (24, 40). However, the widely prescribed antiviral for COVID-19, Paxlovid, was not advised for transplant patients due to significant drug interactions (41). Paxlovid is a combination of SARS-CoV-2 main protease inhibitor nirmatrelvir and cytochrome P450-3A4 inhibitor ritonavir. Ritonavir is found to interact with other drugs such as CsA and tacrolimus to trigger complications. In this regard, combining nirmatrelvir with calcineurin inhibitors such as CsA and VIVIT might help to explore a new combination regimen for organ transplant patients with COVID-19. Calu-3 cells were infected with the SARS-CoV-2 virus and then treated with specified drug combinations and concentrations. With the treatment of increasing dosages of calcineurin inhibitor CsA or VIVIT, a significant reduction in viral titer in Calu-3 cell supernatant was observed (Fig. 9A). Treatment of nirmatrelvir alone resulted in a marked decrease in viral titer (Fig. 9A). Remarkably, a combination of CsA or VIVIT with nirmatrelvir produced a more pronounced reduction in viral titer at 1 µM, indicating profound inhibition of SARS-CoV-2 replication (Fig. 9A). The combinations of CsA + nirmatrelvir or VIVIT + nirmatrelvir brought about an even more prominent drop of viral titer at a low dosage of 0.1 µM (Fig. 9A). Thus, a combination of calcineurin inhibitors with nirmatrelvir might prove useful in certain circumstances.

We then harvested the lysates of infected cells to check for SARS-CoV-2 protein levels. When mock Calu-3 cells were infected with SARS-CoV-2, SARS-CoV-2 N protein was abundantly detected (Fig. 9B, lanes 1 and 2). Treating the infected Calu-3 cells with increasing doses of CsA, VIVIT, or nirmatrelvir resulted in diminution of N protein detected (Fig. 9B, lanes 3–14), reflecting the mitigation of SARS-CoV-2 replication by these drugs. Notably, treating the infected Calu-3 cells with combinations of CsA + nirmatrelvir and VIVIT + nirmatrelvir produced a remarkable drop of N protein in cells than nirmatrelvir alone (Fig. 9B, lanes 15–22 vs 11–14). Although a probable loss of cell lysate might exaggerate the effect to some extent in one group (Fig. 9B, lane 21), the trend of the cooperative inhibition of N protein expression by nirmatrelvir plus CsA or VIVIT was clear (Fig. 9B, lanes 15–22). This suggested that combining calcineurin inhibitors CsA and VIVIT with nirmatrelvir yields potent and synergistic antiviral effect against SARS-CoV-2.

## DISCUSSION

CsA has long been suggested to be a pan-coronavirus inhibitor (22). Its antiviral effects against SARS-CoV, MERS-CoV, HCoV-HKU1, HCoV-229E, and recently SARS-CoV-2 have been documented (42). It has been thought to counteract the replication of coronaviruses including SARS-CoV-2 by blocking viral entry (26, 43, 44), yet the exact mechanism remains unknown. To provide a more thorough analysis of CsA-mediated suppression of SARS-CoV-2 replication, we examined whether CsA-regulated NFAT signaling would contribute to SARS-CoV-2 replication. Our work provides an alternative model to explain the role of Nsp1 in SARS-CoV-2 replication. Nsp1 induces the activation of the cellular NFAT signaling to promote viral replication. Mechanistically, Nsp1 triggers NFAT1 activation by prohibiting the interaction between the inhibitor protein RCAN3 and CnA to substantiate CnA phosphatase activity on NFAT1. The activation of NFAT1 is multifunctional, which activates multiple cellular genes that favor viral replication. As suggested by re-analysis of transcriptomic data from Nsp1-expressing cells and validated with a series of experiments, DDX5 is one of the key NFAT target genes that mediates Nsp1-induced augmentation of SARS-CoV-2 replication.

Our study also provides a detailed mechanism by which NFAT facilitates SARS-CoV-2 replication through activation of CnA instead of cyclophilin A or B. This is consistent with the phenotype of cyclophilin knockout in which an effect on SARS-CoV-2 replication is not seen (26). Nsp1 alleviates RCAN3-mediated regulatory inhibition of CnA to enable NFAT dephosphorylation. The mitigation of RCAN3-mediated negative feedback regulation of calcineurin activity by Nsp1 would then elicit NFAT1 activation to induce the expression of proviral factors including DDX5.

The biological significance of Nsp1-mediated NFAT activation in SARS-CoV-2 replication has been validated using the ΔNsp1 recombinant virus. Enforced expression of CnA, NFAT1, DDX5, and other effector proteins in NFAT signaling resulted in a significant enhancement of ΔNsp1 replication, mimicking NFAT activation during natural infection. Antagonism of the RCAN3-mediated suppression of CnA-induced replication of ΔNsp1 by Nsp1 lent support to the biological role of Nsp1-mediated NFAT activation in SARS-CoV-2 replication. However, even though we showed the importance of Nsp1 to viral replication, the replication kinetics of SARS-CoV-2 and ΔNsp1 might not be fairly compared, as the difference in cellular contexts and replicability of these viruses would be large.

Our functional screening for NFAT-activating SARS-CoV-2 proteins has identified other candidate viral activators of NFAT signaling that merit further analysis. It will also be of interest to determine how Nsp1 might cooperate with the other Nsp proteins to induce NFAT activation in infected cells. SARS-CoV-2 Nsp1 has been previously shown to mediate host translational shutoff and selective activation of viral translation (7). Our findings on its new role in NFAT activation cannot be explained by translation control. In addition, our various rescue experiments have lent critical support to the specificity of the action of Nsp1 and NFAT in SARS-CoV-2-infected cells. However, it is still intriguing to study how Nsp1 could execute its multiple functions in a coordinated fashion during SARS-CoV-2 infection.

Elevated NFAT expression was found in COVID-19 patients (23). This elevation is possibly induced by the positive feedback mechanism of NFAT activation (45). The activation of NFAT would induce NFAT expression through positive feedback and RCAN expression through negative feedback (46). The negative feedback regulation by RCAN would mitigate CnA activity to restore basal NFAT activity. Yet, this negative feedback is disrupted by Nsp1 through the alleviation of RCAN3-mediated suppression of CnA. Thereby, only the positive feedback of NFAT persists, leading to constitutively elevated NFAT expression in COVID-19 patients. Our findings revealed that SARS-CoV-2 usurps cellular regulatory mechanisms on NFAT to induce DDX5. This not only shed light on how SARS-CoV-2 modulates the cellular environment during viral replication but also provided clues to the cell tropism of SARS-CoV-2. SARS-CoV-2 infects and replicates in the upper respiratory tract, intestinal cells, as well as lymphocytes (47). The replicability of the virus in different cell types might be affected by the cellular expression levels of NFAT. The cell types with higher NFAT expression or fewer regulatory proteins on CnA activity could be more susceptible to SARS-CoV-2 infection and replication.

In our study, we also validated that inhibition of CnA-NFAT signaling serves as an effective strategy to antagonize SARS-CoV-2 replication. CsA is known as an immunosuppressant that suppresses the phosphatase activity of CnA to inhibit NFAT activation (48). The drug has also been thought to be a broad-spectrum inhibitor of coronaviruses. Yet, no large-scale clinical trials have been conducted to evaluate its effectiveness in the treatment of COVID-19 due partly to the generic nature of CsA. Immunocompromised COVID-19 patients such as organ transplant recipients taking CsA to minimize graft rejection have often been found to need much longer recovery time, which raises doubt about whether the class of calcineurin inhibitors might have antiviral effects (49, 50). With an in-depth mechanistic analysis on CsA-mediated suppression of SARS-CoV-2 replication, we showed that inhibiting calcineurin activity can mitigate SARS-CoV-2 replication. The longer recovery time of organ transplant patients on CsA could be caused by the prolonged suppression of innate and adaptive immune responses (51). Thus, new antiviral strategies to inhibit coronavirus replication via calcineurin suppression might be designed to combat coronavirus infection. To our surprise, montelukast did not show anti-SARS-CoV-2 activity, although it has been predicted to be an inhibitor of Nsp1 in an *in silico* approach (38) and shown to reverse the cytopathic effect ascribed to Nsp1 (52). Montelukast is a leukotriene receptor antagonist with anti-inflammatory properties (53). Further investigations are required to clarify whether it has any influence on other functions of Nsp1.

Furthermore, we found that a combination of calcineurin inhibitors and SARS-CoV-2 main protease inhibitor nirmatrelvir produced a synergistic antiviral effect in Calu-3 cells. The combinations of CsA + nirmatrelvir and VIVIT + nirmatrelvir resulted in much more pronounced suppression of SARS-CoV-2 replication than CsA, VIVIT, or nirmatrelvir alone. These findings raise the possibility of prescribing nirmatrelvir for organ transplant recipients with COVID-19 who have been on CsA to prevent graft rejection. The approved anti-SARS-CoV-2 drug Paxlovid was previously not recommended for transplant patients in light of the potential drug-drug interaction between ritonavir and calcineurin inhibitor leading to limited effectiveness (41). Based on our findings, it will be of interest to further investigate whether the effectiveness of nirmatrelvir + CsA or nirmatrelvir + VIVIT in viral inhibition and clearance might be fully harnessed *in vivo* in the absence of ritonavir.

**Fig 10:**
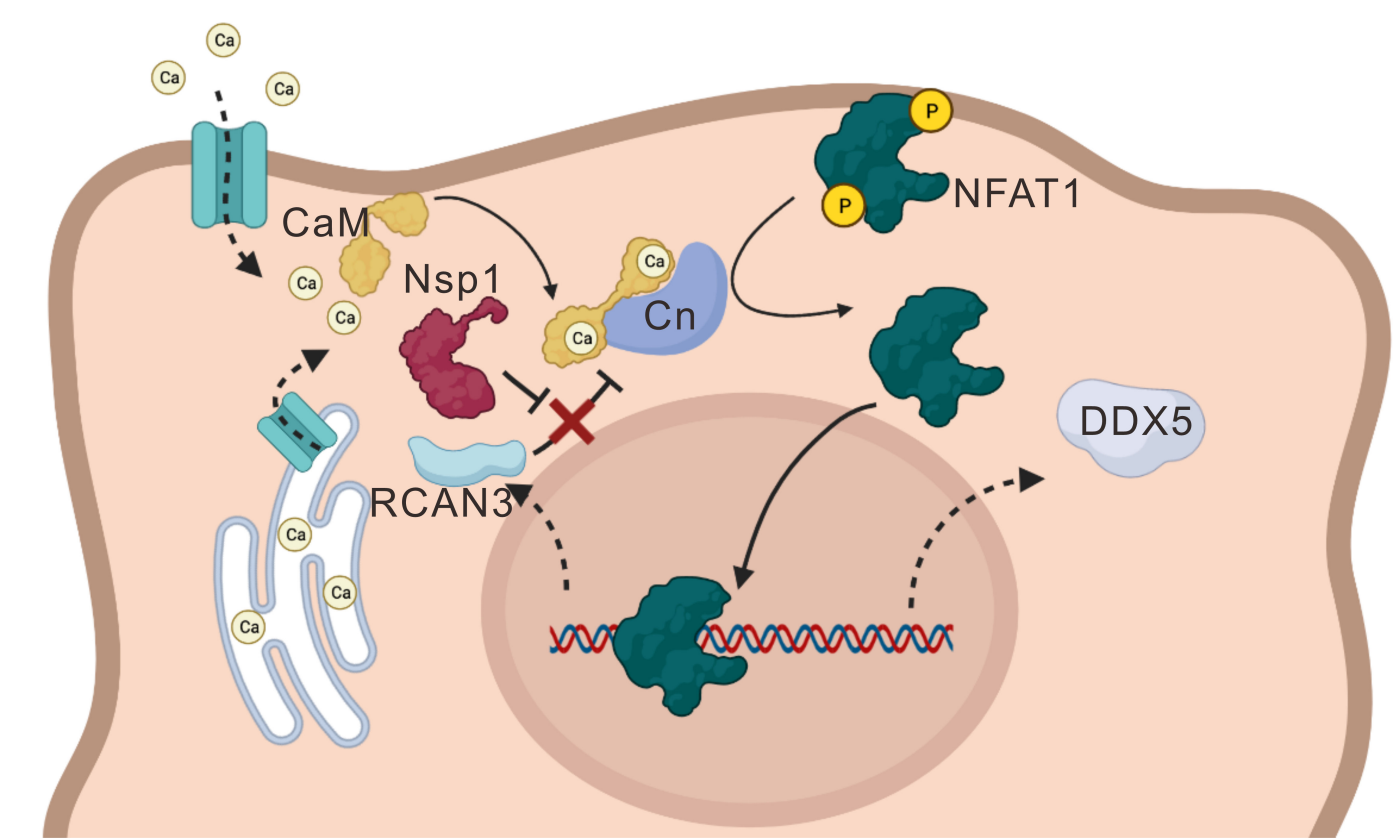
A working model. Influx of Ca^2+^ through ion channel results in the activation of calmodulin (CaM), which interacts with and stimulates calcineurin (Cn) to dephosphorylate and activate NFAT1, leading to its nuclear translocation and activation of target genes including DDX5. RCAN3 is a negative regulator of NFAT that forms a complex with Cn. SARS-CoV-2 Nsp1 interacts with Cn to prevent its interaction with RCAN3, thereby depressing NFAT1.

Taken together, the SARS-CoV-2-encoded Nsp1 was shown to facilitate viral replication by activating the NFAT pathway (Fig. 1). Overexpressing Nsp1 boosted SARS-CoV-2 replication, and enforced expression of CnA and NFAT1 promoted the replication of not only SARS-CoV-2 but also its ΔNsp1 mutant (Fig. 2 to 4). Mechanistically, Nsp1 drove NFAT activation by binding to CnA to impede its interaction with RCAN3 (Fig. 5 and 6). Nsp1 and NFAT1 induced the expression of DDX5, where DDX5 knockdown compromised CnA- or Nsp1-induced potentiation of ΔNsp1 replication (Fig. 7). Whereas CsA and VIVIT exhibited antiviral activity (Fig. 8), their combination with nirmatrelvir produced robust and synergistic anti-SARS-CoV-2 effect (Fig. 9). Our key findings are depicted in Fig. 10.

One major limitation of this study is the lack of loss-of-function experiments for the effector proteins in the NFAT pathway. We have attempted to knock down the expression of CnA or NFAT1 using CRISPR/CasRx (54). However, the knock down of these genes would usually result in a remarkable decrease in the growth rate and viability of the cells. This may be caused by the fact that severe loss of NFAT activity would lead to severe disruption of normal cellular functions, resulting in abnormal cell growth (24). Treatment of cells with a high concentration of CsA (>20 µM) also resulted in severe slowdown of cell growth, indicating that a basal CnA/NFAT function has to be maintained for normal cell functions. Without such a loss-of-function study, most of our analysis on the role of NFAT in SARS-CoV-2 replication relied on gain-of-function experiments and the ΔNsp1 model, which has remedied at least to some extent the absence of loss-of-function data about the influence of NFAT signaling on SARS-CoV-2 biology. The second limitation of our work is the limited viral replicability of the ΔNsp1 virus. Even though we can successfully construct and produce the ΔNsp1 mutant viruses from bacmid, the replicability of the resulting mutant virus was severely impaired in interferon-deficient cells. The mutant ΔNsp1 was much less replicable than the WT SARS-CoV-2 virus. The replication-defective ΔNsp1 virus was mainly detected by RT-qPCR in this study. While the viral N protein of ΔNsp1 was detectable when Nsp1 was coexpressed in cells, the effect of other NFAT effector proteins on SARS-CoV-2 replication can hardly be shown by Western blotting. Meanwhile, the replication-defective ΔNsp1 also failed to form plaques large enough for observation. Therefore, only RT-qPCR can be used to assess the impact of CnA and NFAT on its replication. As suggested by others (19), ΔNsp1 and other Nsp1-defective mutants might serve as a candidate strain for the development of a live attenuated vaccine against SARS-CoV-2. However, the replicability of the mutant viruses has to be sufficiently high for vaccine production. The third limitation of our study is the lack of *in vivo* study for the effectiveness of the drugs and drug combinations of interest. All pilot assays were done in cellular models. Further validation of our findings in hamster and transgenic mouse models as described in references (55, 56) is absolutely required. Nevertheless, our analysis in the human lung cell model might still be informative and instructive for further investigations.

## MATERIALS AND METHODS

### Plasmids

The expression plasmid for FLAG-calcineurin was kindly provided by Dr. Martin Pule, University College London (57). The HA-NFAT1 plasmid was a gift from Prof. Anjana Rao of La Jolla Institute for Immunology, California, USA (58). The SARS-CoV-2 protein expression library was kindly provided by Prof. Kwok-Yung Yuen from the Department of Microbiology, The University of Hong Kong. The Wuhan-Hu-1 strain SARS-CoV-2 bacmid (p-BAC-SARS-CoV-2) was previously described (59).

### Cell culture

Human embryonic kidney cell line HEK293T (CRL-3216) and lung adenocarcinoma cells Calu-3 (HTB-55^Tm^) were purchased from ATCC. Vero-E6-TMPRSS2 cells were purchased from JCRB Cell Bank. Human embryonic kidney cell line HEK293T and Calu-3 cells were cultured in Dulbecco’s modified Eagle medium (DMEM) (Gibco, Life Technologies) supplemented with 10% fetal bovine serum (FBS; Gibco, Life Technologies) and 50 U/mL penicillin-streptomycin (Pen-Strep; Thermo Fisher) at 37°C in a 5% CO_2_ atmosphere. Vero-E6-TMPRSS2 cells were cultured in DMEM supplemented with 10% FBS, 1% Pen-Strep, and 1 mg/mL G418 [G418 (Geneticin); InvivoGen].

### Transient transfection

HEK293T cells were seeded onto 60-mm dishes or 24-well plates (Iwaki) at a concentration of 1 × 10^5^ per mL and transfected with GeneJuice (Novagen) 24 hours later in a 1 µg DNA to 3 µL GeneJuice ratio. Vero-E6-TMPRSS2 and Calu-3 cells were seeded onto the 6-, 12-, 24-, or 96-well plates at a concentration of 2 × 10^5^ per mL and transfected with Lipofectamine3000 (Thermo Fisher) 24 hours post-seeding in a ratio of 1 µg DNA to 3 µL Lipofectamine3000.

### Protein analysis

Dual luciferase reporter assay, co-immunoprecipitation, and Western blotting were performed as described (60).

### RT-qPCR

The RNA samples were first treated with DNase I [1 µL Ambion DNase I (Invitrogen), 2 µL Ambion 10× DNase I buffer (Invitrogen), 8 µg RNA, and DEPC H_2_O for topping up the total volume to 20 µL] for incubation at 37°C for 30 min followed by 20 min heat inactivation at 65°C. A volume of 10 µL of the treated RNA was added with 3 µL of 10 µM primer (random hexamer, oligonucleotides, or specific primers) and incubated at 65°C for 10 min. The solution was then added with 4 µL of 5× reverse transcriptase buffer (Roche), 0.5 µL of Protector RNase Inhibitor (Roche), 2 µL of deoxynucleotide (Roche), and 0.5 µL of reverse transcriptase (Roche) for cDNA synthesis. The RT program was 55°C for 30 min, 85°C for 5 min, and followed by 4°C. The cDNA samples were then stored at −20°C. The transcript levels of NFAT, SARS-CoV-2 RdRp, Nsp1, dsRED, Sox2, JAG1, DDX5, Bcl3, Foxo1, and Xbp1 were determined by SYBR Premix Ex Taq II (Tli RNase H Plus) (TaKaRa) in StepOne Real-Time PCR System (Thermo Fisher). The transcript levels were normalized with glyceraldehyde 3-phosphate dehydrogenase (GAPDH) mRNA level. Sequences of qPCR primers were SARS-CoV-2 RdRp (forward 5′-ATGAGCTTAG TCCTGTTG and reverse 5′-CTCCCTTTGT TGTGTTGT), Nsp1 (forward 5′-AGCCTTGTCC CTGGTTTCAA and reverse 5′-ACGTGCCTCT GATAAGACCT), dsRED (forward 5′-AAGGTGAAGT TCATCGGCGT and reverse 5′-TTGTGGATCT CGCCCTTCAG), Sox2 (forward 5′-GCTACAGCAT GATGCAGGAC CA and reverse 5′-CCAAACATAA ATGCCCCATC), JAG1 (forward 5′-TGCTACAACC GTGCCAGTGA CT and reverse 5′-TCAGGTGTGT CGTTGGAAGC CA), DDX5 (forward 5′-CGCAGTACCA AAACAGGCAC and reverse 5′-AGTATCTGTC CCGACGGTCA), Bcl3 (forward 5′-CTCATCCACG CCGTGGAAAA C and reverse 5′-TCAGCTGCCT CCTGGAGCTG G), Foxo1 (forward 5′-CTACGAGTGG ATGGTCAAGA GC and reverse 5′-CCAGTTCCTT CATTCTGCAC ACG), Xbp1 (forward 5′-CAGACTACGT GCACCTCTGC and reverse 5′-CTGGGTCCAA GTTGTCCAGA AT) and GAPDH (forward 5′-AGAAGGCTGG GGCTCATTTG and reverse 5′-CTGTGGTCAT GAGTCCTTC).

### BAC recombineering

Procedures of BAC recombineering have been described previously (60). Here, two galK primers with 50 bp homology arm flanking the desired mutation site were designed (forward 5′-GATCATCAGC ACATCTAGGT TTCGTCCGGG TGTGACCGAA AGGTAAGATG CCTGTTGACA ATTAATCATC GGCA and reverse 5′-GGGTAGCCAT CAGGGCCACA GAAGTTGTTA TCGACATAGC GAGTGTATGC TCAGCACTGTCC TGCTCCTT). Another pair of oligos were designed with 100-bp complementary oligos with the deletion of Nsp1 (ΔNsp1 forward 5′-GATCATCAGC ACATCTAGGT TTCGTCCGGG TGTGACCGAA AGGTAAGATG GCATACACTC GCTATGTCGA TAACAACTTC TGTGGCCCTG ATGGCTACCC and reverse 5′- GCATACACTC GCTATGTCGA TAACAACTTC TGTGGCCCTG ATGGCTACCC CATCTTACCT TTCGGTCACA CCCGGACGAA ACCTAGATGT GCTGATGATC). The generation of dS-dsRED replicon was based on dS-galk, which was described previously (30). dsRED coding sequence with 50-bp homology arm targeting the galk region of dS-galk was PCR amplified by primer: forward 5′- CAACAGAGTT GTTATTTCTA GTGATGTTCT TGTTAACAA CTAAACGAAC AATGGCCTCC TCCGAGG and reverse 5′-AGTTACAGTT CCAATTGTGA AGATTCTCAT AAACAAATCC ATAAGTTCGT TTATCTAGAT CCGGTGGATC CCG. The resulting PCR fragment was used as the raw insert to generate dS-dsRED through BAC recombineering described (60).

### SARS-CoV-2 virus production

A total of 2 × 10^5^/mL VeroE6-TMPRSS2 or HEK293T cells were seeded onto a T75 culture flask (Thermo Fisher). Ten micrograms of pBelo-SARS-CoV-2 bacmid was transfected to each flask of cells 24 hours after the cells were seeded. Four days post-transfection, the culture medium of the flask was centrifuged, and the supernatant was collected.

### SARS-CoV-2 infection and plaque assay

The newly produced or passaged SARS-CoV-2 viruses were first quantified by plaque assay. A total of 3 × 10^5^/mL VeroE6-TMPRSS2 cells were seeded onto a 6-well plate, and the virus medium was serially diluted. A volume of 100 µL of the serially diluted viral cultures was added to VeroE6-TMPRSS2 cells and incubated for 1 hour. After incubation, 1% agar in DMEM overlay was added to each well. The infected cells were incubated at 37°C for 2 days. After 2 days, 4% paraformaldehyde was added to each well for overnight incubation. After incubation with paraformaldehyde, the solid overlay was removed, and the cells were stained with 0.5% crystal violet solution (0.5 g crystal violet powder in 100 mL of 20% ethanol solution) for 10 min. The crystal violet solution was then removed, and the stained cells were rinsed with water. The plaque-forming units were counted.

For SARS-CoV-2 infection experiments to harvest protein samples or conduct drug treatment, 3 × 10^5^/mL VeroE6-TMPRSS2 or Calu-3 cells were seeded onto 6-, 12-, or 24-well plates. The cells would then be infected with desired M.O.I. of viruses for 1–1.5 hours. After incubating with the virus culture, the medium would be changed to DMEM (with 2% FBS) or to DMEM supplemented with the desired drug at a specific concentration, and the cells would be incubated at 37°C for 2 days (for VeroE6- TMPRSS2) or 3–4 days (for Calu-3 cells). After incubation, the protein samples would be harvested by incubating the cells with RIPA solution (with 1× protein sample buffer) for 30 min. Then, the samples were transferred out of the hood and boiled for 10 min before being taken out of the Biosafety Level 3 Physical Containment Facility.

### Nanopore sequencing

The viral RNA samples harvested by QIAamp viral RNA minikit (QIAGEN) were sequenced by nanopore sequencing (Oxford Nanopore Technologies) using Oxford Nanopore MinION device R9.4.1 flow cell according to the manufacturer’s instructions. Bioinformatic analysis was performed with the ARTIC-nCoV network workflow (61).

### Transmission electron microscopy

The procedure of TEM was described previously (30). In short, viruses were fixed with 4% paraformaldehyde for 30 min. The fixed viruses were negatively stained with uranyl acetate. A volume of 10 µL of the fixed virus was mounted onto a hydrophilized formvar-carbon-coated copper grid by grid floating. The grid was then immersed in 10 µL of 2% uranyl acetate for 1 min. The grid was then washed with ddH_2_O droplet and excess liquid was removed by filter paper. The grid was then dried by air and examined with FEI TeCnai G2 20 scanning TEM at 100 kV (32).

## Data Availability

All data reported in this paper will be shared by the lead contact upon request.
